# A Genome-Wide Characterization of Receptor-like Cytoplasmic Kinase IV Subfamily Members in *Populus deltoides* Identifies the Potential Role of *PdeCRCK6* in Plant Osmotic Stress Responses

**DOI:** 10.3390/plants13233371

**Published:** 2024-11-30

**Authors:** Huanhuan Pan, Zhengquan He, Linxiu Liu, Renyue Cai, Hu Huang, Xinru Xie, Xun Cao, Yanan Li, Wenmin Qiu, Zhuchou Lu, Xiaojiao Han, Guirong Qiao, Renying Zhuo, Jianjun Hu, Jing Xu

**Affiliations:** 1 Key Laboratory of Three Gorges Regional Plant Genetic & Germplasm Enhancement (CTGU), Biotechnology Research Center, China Three Gorges University, Yichang 443002, China; phuanhuan2023@163.com (H.P.); zhq_he@163.com (Z.H.); 16671050824@163.com (X.C.); 2 State Key Laboratory of Tree Genetic and Breeding, The Research Institute of Subtropical Forestry, Chinese Academy of Forestry, Hangzhou 311400, China; lxliu9968@163.com (L.L.); 15272978041@163.com (H.H.); xie2676527718@163.com (X.X.); l160915013@163.com (Y.L.); qiuwm05@163.com (W.Q.); sluzc@caf.ac.cn (Z.L.); hanxj@caf.ac.cn (X.H.); gr_q1982@163.com (G.Q.); zhuory@gmail.com (R.Z.); 3 Tiantai Forestry Technology Promotion Station, Tiantai 317200, China; cairenyue@126.com; 4 State Key Laboratory of Tree Genetics and Breeding, Key Laboratory of Tree Breeding and Cultivation of National Forestry and Grassland Administration, Research Institute of Forestry, Chinese Academy of Forestry, Beijing 100091, China

**Keywords:** *Populus deltoides*, RLCK-IV subfamily, CRCK, osmotic stress, gene family

## Abstract

The IV subfamily of receptor-like cytoplasmic kinase (RLCK-IV), known as calcium-binding receptor-like cytoplasmic kinases (CRCKs), plays a vital role in plant signal transduction, particularly in coordinating growth and responses to abiotic stresses. However, our comprehension of CRCK genes in *Populus deltoides*, a species characterized as fast-growing and pest-resistant but with drought intolerance, is limited. Here, we identify 6 members of the CRCK subfamily on a genome-wide scale in *P. deltoides*, denoted as *PdeCRCK1*–*PdeCRCK6*. An evolutionary and structural analysis revealed highly conserved kinase catalytic domains across all PdeCRCKs, characterized by calmodulin (CaM)-binding sites and serine (Ser)/threonine (Thr) phosphorylation sites. The *cis*-acting elements of promoters indicated the presence of responsive elements for plant hormones, abiotic stresses, and transcription factor binding sites, which is supported by the distinct transcriptional expression patterns of *PdeCRCKs* under abscisic acid (ABA), polyethylene glycol (PEG), and mannitol treatments. A transient overexpression of *PdeCRCK3/5/6* in tobacco (*Nicotiana benthamiana*) leaves indicated their involvement in reactive oxygen species (ROS) scavenging, polyamine gene synthesis, and ABA signaling pathway modulation. Immunoprecipitation–Mass Spectrometry (IP–MS) and a yeast two-hybrid (Y2H) assay showed that PdeCRCK6 interacted with AAA-type ATPase proteins and ubiquitin, suggesting its potential function in being involved in chloroplast homeostasis and the 26S ubiquitin protease system. Taken together, these findings offer a comprehensive analysis of the RLCK-IV subfamily members in *P. deltoides*, especially laying a foundation for revealing the potential mechanism of *PdeCRCK6* in response to osmotic stresses and accelerating the molecular design breeding of drought tolerance in poplar.

## 1. Introduction

Poplar, a crucial industrial wood resource known for its rapid growth and high wood yield, is widely distributed in the arid and semi-arid regions of the northern hemisphere [1]. Extreme climate changes have led to an increase in the frequency of droughts, significantly adversely affecting poplar growth and development, forest productivity, and quality and even leading to large dead areas. Cultivating new germplasm with enhanced drought tolerance and high yields is of great practical importance for poplar production. Identifying and characterizing the drought-responsive genes and mechanisms in poplar using a bioinformatic analysis and expression pattern analysis followed by preliminary functional validation will provide valuable insights for the cultivation of drought-tolerant poplar species via genetic improvements.

Plants encounter drought stress and rapidly respond to osmotic stress. Receptor-like kinases (RLKs) are conserved protein kinases on a plant cell membrane that transduce external signals to the nucleus of plant cells and affect plant growth, development, stress responses, and disease resistance. Upon detecting abiotic stress, RLKs undergo autophosphorylation or interact with other proteins, triggering the activation of downstream signaling cascades. The activation of these signaling pathways ultimately leads to the expression of stress-responsive genes, which can code for proteins that help in osmotic adjustments, protect cellular structures, and enhance tolerance to water deficits. RLKs consist of an amino-terminal extracellular domain (ECD), a transmembrane (TM) domain, and an intracellular kinase domain, i.e., Pkinase (pfam00069) and Pkinase-Try (pfam07714) [2,3,4]. Variable ECDs can be classified into 15 RLK subfamilies, including the leucine-rich repeat receptor (LRR), lectin (C-Lec and L-Lec), LysM-RLKs, cysteine-rich receptor-like kinases (CRKs), and receptor-like cytoplasmic kinase (RLCK), etc., which have been identified in various plant species in response to abiotic stresses [3,5,6,7]. LRR-RLKs represent the largest subfamily of RLKs playing diverse functions in plants. In *Setaria*, DROOPY LEAF1 (DPY1), a plasma-membrane (PM)-localized LRR-RLK, acts as a sensor and functions as a sensor and acts upstream of the activation of STRESS-ACTIVITED PROTEIN KINASES (SAPK6, a subclass of I SnRK2), which is essential for the acclimation of foxtail millet to drought stress [8]. In *Arabidopsis thaliana*, the LRR-RLK gene *HSL3* negatively regulates drought tolerance by controlling abscisic acid (ABA) signaling pathways and modulating H_2_O_2_-mediated stomatal closures [9]. Lec-RLKs also form a large subfamily of RLKs, which include three types of lectins, G, L, and C lectin. Presently, Lec-RLKs have been identified in *A. thaliana* [10], *cucumis sativus* [11], *Vigna radiata* [5], peanut plants (*Arachis hypogaea*) [12], and wheat (*Triticum aestivum*) [13]. CRK is another cysteine-rich repeat domain kinase receptor that contains C-X_8_-C-X_2_-C motifs [14]. In *A. thaliana*, an overexpression of *CRK5* confers higher drought tolerance, and *CRK5* may mediate ABA signaling through catalyzing the phosphorylation of downstream targets by the cytoplasmic kinase domain [15]. In potatoes (*Solanum tuberosum*), ten CRLKs (cysteine-rich receptor-like kinases) have been identified. *StCRLK9* has been identified as a potential candidate that responds to heat, salt, and drought stress [16]. Remarkably, several subfamilies of the RLK gene family have been reported in wheat, including LRR-RLK [17], Lys-M RLK [18], receptor-like protein kinase 1 (RPK 1) [19], CRK [20], etc. TaCRK10 acts as an important sensor of *Puccinia striiformisf*. sp. *Tritici* (*Pst*) infection and high temperatures [6]. *TaPRK2697* directly interacts with *TaSR* and enhances Na^+^ uptake, thereby increasing salt tolerance in wheat [21]. *TaRPK1* displays a higher expression in drought-tolerant varieties than drought-susceptible varieties [19]. The ortholog of wheat LRK10 in *A. thaliana*, AtLRK10L1.2, acts as a positive regulator in response to drought tolerance by closing stomata, which may occur either directly or indirectly via ABA-mediated signaling [22].

Receptor-like cytoplasmic kinases (RLCKs), a subfamily of RLKs lacking an extracellular domain, play major roles in plant growth and development, responses to biotic/abiotic stresses, and the regulation of endogenous extracellular signaling molecules by modulating cell activities [3,23,24]. Most RLCK proteins are characterized by a Ser/Thr intracellular kinase domain, while some RLCKs also contain additional domains, such as U-box, leucine-rich repeat sequences (LRRs), pentapeptide repeat sequences (PPRs), WD40, and epidermal growth factor (EGF) domains [23]. RLCKs, or their subfamily, have been identified in *A. thaliana*, rice [10,23], wheat [13], maize (*Zea mays*) [14], *Gossypium hirsutum* and upland cotton [15,16], and cassava (*Manihot esculenta Crantz*) [25].

The *A. thaliana* RLCK member ARCK1 (ABA-AND OSMOTIC-STRESS-INDUCIBLE RECEPTOR-LIKE CYTOSOLIC KINASE 1) interacts with and is phosphorylated by AtCRK36 to form a complex that functions as a negative regulator in the ABA and osmotic stress signaling pathways [26]. In *A. thaliana*, calcium/calmodulin-regulated receptor-like kinases (CRLKs) 1 and 2, which belong to the RLCK-IV subfamily, have been observed to inhibit the cold-induced activation of MPK3/6, which is intricately associated with the negative regulation of a cold stress response [27,28]. Similarly, in rice, the RLCK protein SALT TOLERANCE RECEPTOR-LIKE CYTOPLAS-MIC KINASE 1 (STRK1) interacts with and enhances catalase activity by phosphorylating CatC, thereby positively regulating responses to salt and oxidative stress [29]. Meanwhile, *GROWTH UNDER DROUGHT KINASE* (*GUDK*, VII-6), a drought-inducible receptor-like cytoplasmic kinase, positively regulates plant drought tolerance and yields by phosphorylating and activating APETALA2/ETHYLENE RESPONSE FACTOR 37 (OsAP37) [30]. In soybeans, *GsCBRLK* (Ca^2+^/CaM-binding RLK, an RLCK-IV member) overexpression increases the stress tolerance of transgenic Arabidopsis [11,27].

Calcium (Ca^2+^) ions act as a key secondary messenger, with calmodulin being an important Ca^2+^ binding protein [31,32]. Calmodulin consists of two similar domains, each containing two EF-hand motifs responsible for specifically binding Ca^2+^ [33]. Calmodulin’s binding sites typically consist of specific sequence motifs, the most common of which is the IQ motif, which is rich in hydrophobic amino acids and is capable of interacting with the C-terminal domain of calmodulin [33]. In *A. thaliana*, a helical wheel projection of the peptide sequences indicates that the amino acids 160–183 in the vicinity of subdomain II possess the structural characteristics necessary for CaM binding. Notably, a hydrophobic residue, Ile172, is embedded within a context of basic residues, Lys173 and Arg174, which are common features observed in known CaM targets [34]. Calcium-binding receptor-like cytoplasmic kinases are essential for maintaining cellular homeostasis in Ca^2+^ signaling. Early studies on RLCK-IV mainly focused on the function of a single gene, and there has been no comprehensive analysis of members of the RLCK-IV subfamily, whose genes encode calcium-binding receptor-like cytoplasmic kinases, known as CRCKs [34]. *P. deltoides*, classified within a section of *Aigeiros*, is a species of significant ecological and economic importance, noted for its rapid growth, disease resistance, moderate genome size (approximately 431 Mb), and ease of experimental manipulation [35,36]. Given the significant roles of RLCK-IV proteins on abiotic stresses in Arabidopsis and soybeans, it is hypothesized that they also play a crucial role in poplar.

Here, a comprehensive genome-wide analysis of RLCK-IV subfamily genes in *P. deltoides* was conducted. Phylogenetic relationships, gene structure, conserved domains and motifs, a collinearity analysis, and *cis*-acting elements were examined. The expression patterns of RLCK-IV genes under various osmotic stresses and ABA treatment conditions were examined. The potential interacting proteins of stress-responsive genes were explored in tobacco leaves. A further exploration of the potential interacting proteins of PdeCRCK6 in *P. deltoides* was conducted through Immunoprecipitation–Mass Spectrometry (IP–MS) and yeast two-hybrid (Y2H) assays. This study aims to provide valuable insights into the molecular mechanisms underlying the role of the RLCK-IV subfamily in the response to droughts in poplars and may contribute to the breeding of resistant poplar cultivars.

## 2. Results

### 2.1. Identification of RLCK-IV/CRCK Subfamily Members in P. deltoides

In this study, six RLCK-IV/CRCK subfamily genes were identified in *P. deltoides* and were designated as *PdeCRCK1* to *PdeCRCK6*, according to their chromosomal position and annotations, as shown in Table 1. The coding sequences of the six *PdeCRCKs* ranged from 1171 bp (*PdeCRCK1*) to 1542 bp (*PdeCRCK6*). The encoded proteins consisted of 389 to 513 amino acids, with molecular weights ranging from 43.623 to 57.581 kDa. The isoelectric points were in the range from 8.78 (PdeCRCK1) to 9.49 (PdeCRCK4), while the instability index varied from 38.31 (PdeCRCK6) to 50.25 (PdeCRCK2). The aliphatic index varied from 76.7 (PdeCRCK2) to 93.1 (PdeCRCK6). Most of the PdeCRCK proteins were predicted to be localized in the nucleus, with some also being predicted to be associated with the cell membrane (Table 1).

**Figure 1:**
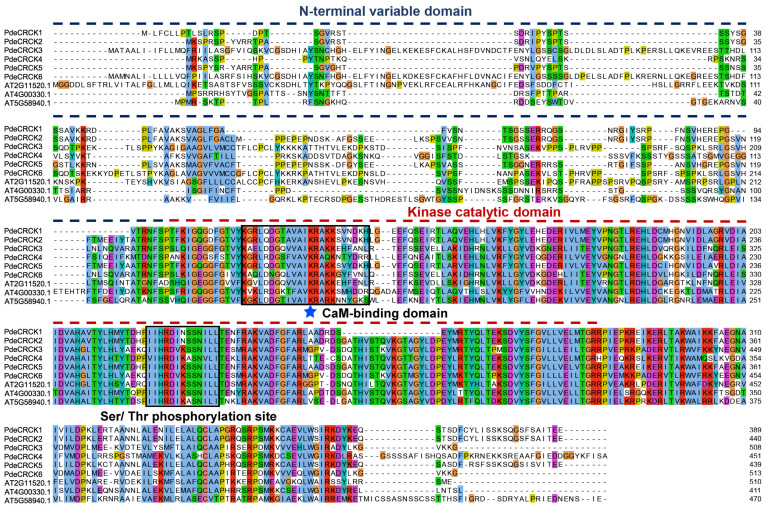
Sequence alignment of the conserved domain of RLCK-IV/CRCK subfamily proteins between *Populus deltoides* and *Arabidopsis thaliana*. The conserved kinase catalytic domain was analyzed in all PdeCRCKs. The black boxes indicate the conserved CaM-binding domain and Ser/Thr phosphorylation site. The blue line indicates the N-terminal variable domains among PdeCRCKs. The important residue Lysine (K) of the CaM-binding domain is highlighted by a blue star.

An examination of the PdeCRCK protein sequences revealed significant conservation of the kinase catalytic domain, which includes the CaM-binding domain and the Ser/Thr phosphorylation site. These domains are crucial for modulating the interaction between the calcium/calmodulin sensor protein and protein kinase activity (Figure 1). In contrast to the conserved calcium-binding domain, the N-terminal variable domains among PdeCRCK proteins exhibit considerable divergence, suggesting potential functional differences in PdeCRCK proteins (Figure 1).

### 2.2. Phylogenetic Analysis of RLCK-IV/CRCKs

**Figure 2:**
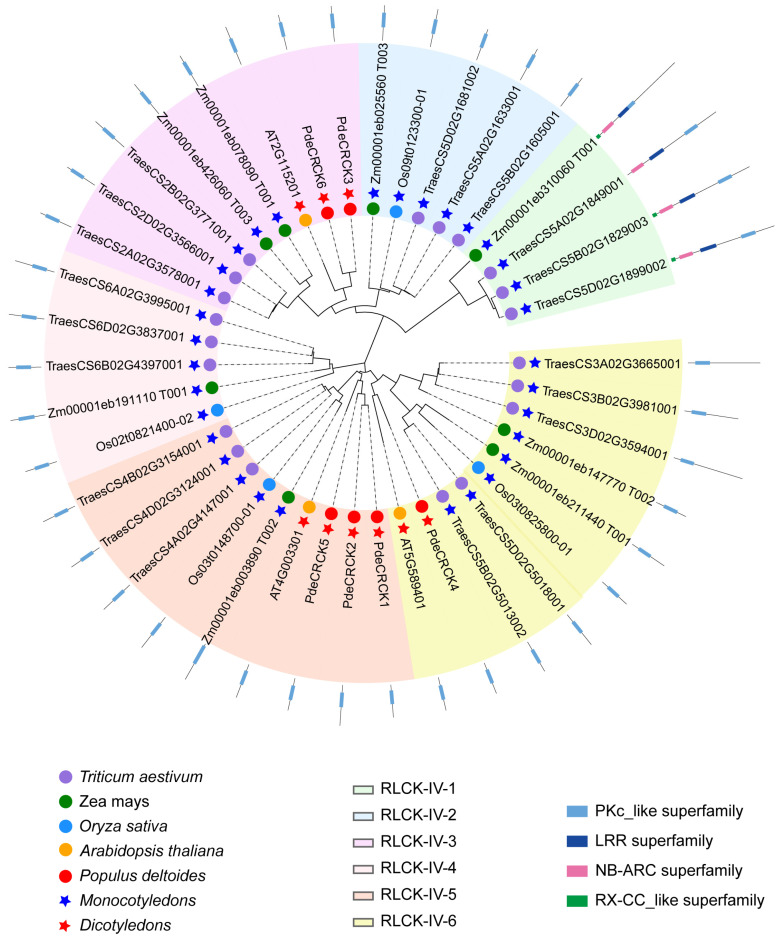
Phylogenetic analysis of green-plant RLCK-IV/CRCK subfamily members. A total of 41 RLCK-IVs were identified in *A. thaliana*, rice, wheat, maize, and *P. deltoides*. The phylogenetic relationship between *P. deltoides* and the aforementioned species was analyzed using the maximum likelihood method (ML) with JTT+G and a bootstrap analysis with 1000 replicates in MEGA 11.0.13. The 41 RLCK-IVs were divided into six groups and designated as RLCK-IV-1 to RLCK-IV-6. The different colors in the circle indicate the different species. The red star represents the dicot plants, while the blue star represents the monocot plants. The rectangles of different colors outside the circle represent different structural domains.

To investigate the genetic evolutionary relationship of the RLCK-IV subfamily among *P. deltoides* and various monocot and dicotyledonous plant species, the RLCK-IV subfamily genes from these six species were employed to construct a phylogenetic tree (Figure 2). The results reveal the classification of 41 members into six groups, designated as RLCK-IV-1 to RLCK-IV-6. Notably, RLCK-IV-1 contained additional LRR, NB-ARC, and RX-CC-like superfamily domains compared to the other five groups. This may have resulted from domain degradation due to functional redundancy during evolution, leading to the retention of the PKc-like superfamily domain, which plays a pivotal role in the RLCK family. Furthermore, it was observed that the members of the RLCK-IV subfamily from dicot and monocot plants cluster distinctly. Specifically, PdeCRCK3, PdeCRCK6, and AT2G115201 form a closely related cluster, indicating a closer phylogenetic relationship among them (Figure 2). Similarly, PdeCRCK1, PdeCRCK2, PdeCRCK5, and AT4G003301 were closely aligned, while PdeCRCK4 was closely aligned with AT5G589401 (Figure 2). Notably, the number of RLCK-IV subfamily members in monocot plants significantly exceeds that in dicot plants, possibly reflecting specific evolutionary adaptations in monocot plants.

### 2.3. Gene Structure and Conserved Motifs of PdeCRCKs

**Figure 3:**
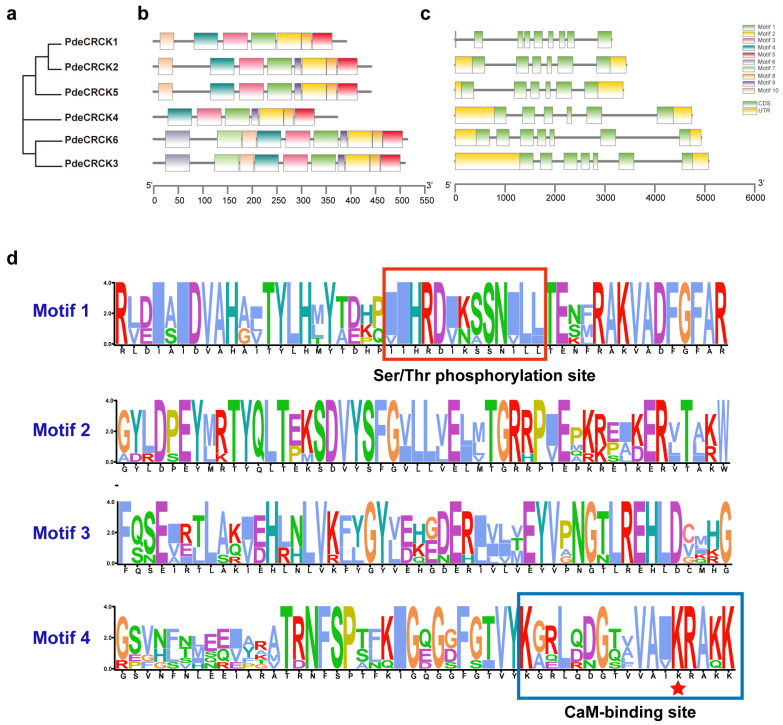
Structural organization of PdeCRCKs. (**a**) A phylogenetic tree that was generated using MEGA 11.0.13. (**b**) The conserved motifs of PdeCRCKs, with different motifs represented by different colors. (**c**) The exon/intron structure of the putative PdeCRCKs, with the yellow boxes indicating exons and green boxes indicating the 3′ or 5′ UTRs (untranslated regions). (**d**) Visualization of conserved motifs by WebLogo, with the red box representing the Ser/Thr phosphorylation site; the blue box representing the CaM-binding site; and the important residue, Lysine (K), of the CaM-binding site is highlighted by a red star.

The phylogenetic tree of the PdeCRCK subfamily expansion was constructed using MEGA 11.0.13 (Figure 3a). A gene structure analysis revealed that PdeCRCKs contain six to nine exons. Specifically, *PdeCRCK1* has the most introns (nine), while *PdeCRCK2*, *PdeCRCK3*, and *PdeCRCK4* each have six, and *PdeCRCK5* and *PdeCRCK6* have seven introns (Figure 3c). Overall, the similar gene structures of all *PdeCRCKs* suggest that they may have related functions.

The conserved motifs within PdeCRCKs were analyzed with the motif number set to ten. The results indicate that PdeCRCK6 and PdeCRCK3 contained ten motifs, whereas PdeCRCK2 and PdeCRCK5 contained eight motifs, excluding motifs 6 and 7. PdeCRCK1 comprised seven motifs, excluding motifs 6, 7, and 9. PdeCRCK4 lacked motifs 6, 7, and 10 (Figure 3b). Overall, genes within the same subgroup exhibited comparable gene sizes and structures, suggesting potential functional similarities in plant growth and development. Additionally, WebLogo was used to visualize conserved motifs, revealing the presence of Ser/Thr phosphorylation sites in motif 1 and the CaM-binding domain in motif 4, which aligns with the results of sequence alignment (Figure 3d).

### 2.4. Chromosome Distribution and Collinearity Analysis of PdeCRCKs

**Figure 4:**
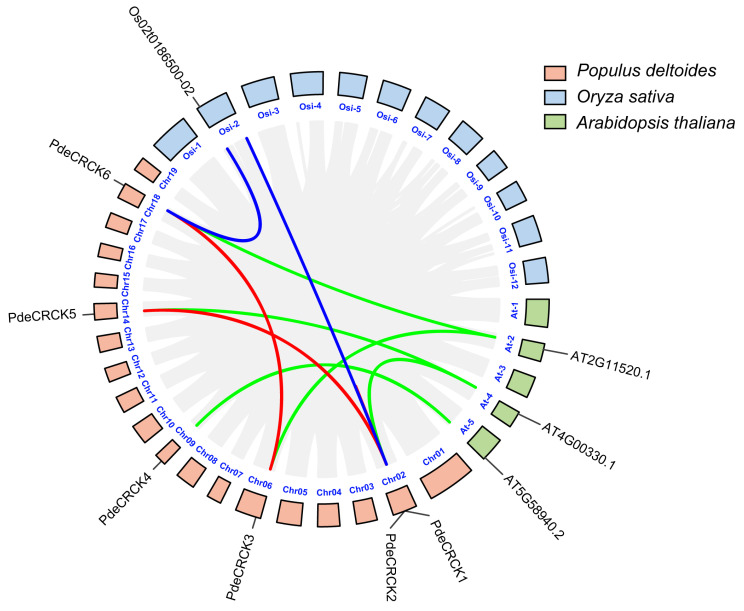
Synteny relationships of gene pairs. The relationships of RLCK-IV duplicated genes between *P. deltoides* and *A. thaliana* are indicated by green lines, between *P. deltoides* and rice by blue lines, and between *P. deltoides* and *P. deltoides* by red lines.

Based on the genomic locations of the six *PdeCRCKs* on their respective chromosomes, the chromosomal distribution of *PdeCRCKs* was mapped onto five chromosomes. Specifically, *PdeCRCK1* and *PdeCRCK2* are located on chromosome 1; *PdeCRCK3* on chromosome 6; and *PdeCRCK4*, *PdeCRCK5*, and *PdeCRCK6* are located on chromosomes 9, 14, and 18 (Figure 4).

Rice and *A. thaliana* are extensively studied model species with well-characterized genomes. A synteny analysis was conducted using TBtools 2.0 to explore the duplication events of RLCK-IVs among *P. deltoides*, *A. thaliana*, and rice. The analysis revealed that *P. deltoides* contain four pairs of PdeCRCKs that show collinearity. Collinearity was observed in two pairs of RLCK-IVs between *P. deltoides* and rice and six pairs between *P. deltoides* and *A. thaliana* (Figure 4). The analysis indicated that the synteny between *P. deltoides* and *A. thaliana* is stronger than that between *P. deltoides* and rice, implying a closer genetic relationship between *P. deltoides* and *A. thaliana*.

### 2.5. Cis-Acting Regulatory Element Identification in Promoter of PdeCRCKs

**Figure 5:**
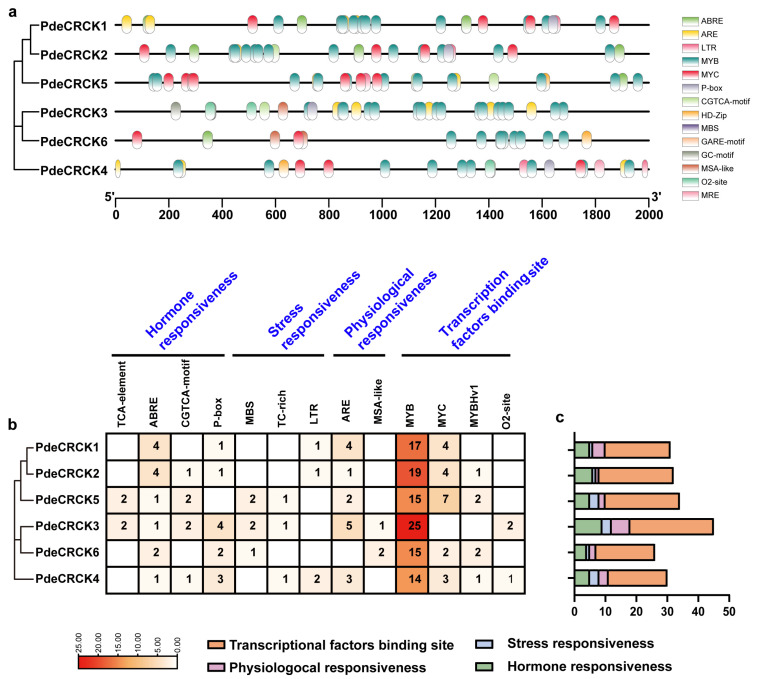
*Cis*-element analysis associated with abiotic stress and phytohormone responsiveness in the promoter regions of *PdeCRCKs*. The 2000 bp region upstream of the transcriptional start site in *PdeCRCKs* was obtained and used to analyze the responsive *cis*-elements. (**a**) The types and distribution of *cis*-elements in the promoters. (**b**) This heatmap shows the number of *cis*-elements, with higher numbers represented in red and lower numbers in white. (**c**) All *cis*-elements were categorized into four groups, and their numbers were counted. Orange represents transcription factor binding sites, blue represents stress-responsive elements, pink represents physiological-responsive elements, and green represents hormone-responsive elements.

To investigate the biological functions and regulatory mechanisms of the six *PdeCRCKs*, a 2000 bp region upstream of the transcriptional start site was analyzed using PlantCARE. Various regulatory elements were identified, including the plant hormone metabolism (ABA, methyl jasmonate (MeJA), gibberellin (GA), and auxin); responses to abiotic stresses (light, droughts, and low temperatures); MYB transcription factor binding sites; and others. The distribution and abundance of *cis*-acting regulatory elements were examined across the six *PdeCRCKs*. Notably, ABRE motifs were present in *PdeCRCKs*, wherein *PdeCRCK1* and *PdeCRCK2* each contained four ABRE elements; *PdeCRCK6* contained two ABRE elements; and *PdeCRCK3*, *4*, *5* each contained one ABRE element (Figure 5b). Additionally, multiple *cis*-elements were responsive to salicylic acid (SA), MeJA, and GA. Furthermore, several other *cis*-elements, including transcription factor binding sites, cell cycle responsive elements, and anaerobic-related elements, were all detected in *PdeCRCKs* (Figure 5a). Overall, these results suggest that *PdeCRCKs* may be involved in responses to hormones and environmental stresses, as evidenced by the presence of multiple *cis*-acting regulatory elements associated with these genes (Figure 5).

### 2.6. Expression Profiles of PdeCRCKs Under Polyethylene Glycol (PEG), Mannitol, and ABA Stresses

**Figure 6:**
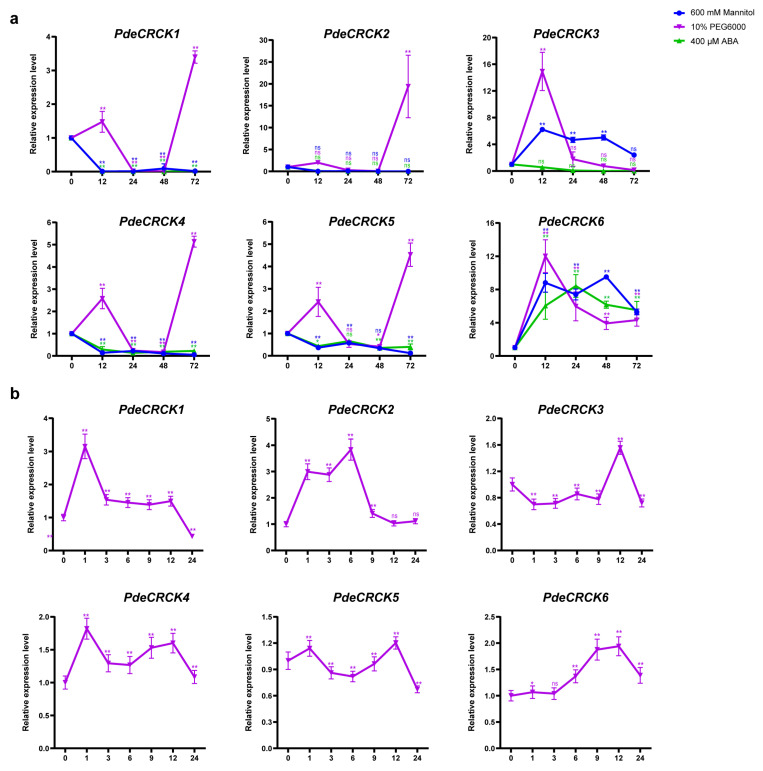
Expression patterns of *PdeCRCKs* responding to various stresses. (**a**) The relative expression levels of *PdeCRCKs* were analyzed based on the leaves of *P. deltoides* under treatments within 72 h. (**b**) The relative expression level of *PdeCRCK* genes were analyzed based on the leaves of *P. deltoides* treated with 10% PEG-6000 within 24 h. For each time point, the expression of *PdeCRCKs* in seedlings without any stress was regarded as a reference. Quantitative analyses of all the measurements were conducted using GraphPad Prism 8. Data are presented as a mean ± standard deviation of three biological replicates. Statistical significance was assessed using Student’s *t*-test and Duncan’s multiple range test: ^ns^ *p* > 0.05, * *p* < 0.05, ** *p* < 0.01.

Following PEG treatment, the transcripts of *PdeCRCK1, PdeCRCK2, PdeCRCK4*, and *PdeCRCK5* initially increased at 12 h, followed by a decline at 24 h and 48 h, and rose sharply again at 72 h (Figure 6a). In contrast, their expression levels consistently decreased under mannitol and ABA treatments. Notably, the transcripts of *PdeCRCK3* initially increased approximately 14.9-fold at 12 h under PEG treatment, subsequently declining to 0.02 times the initial level by 72 h (Figure 6a). Meanwhile, under mannitol treatment, *PdeCRCK3* transcripts initially increased by 6.22-fold, exhibiting fluctuations thereafter, and reached 2.39 times the initial level by 72 h (Figure 6a). In contrast, under ABA treatment, *PdeCRCK3* transcripts continuously decreased, reaching 0.01 times the initial level by 72 h. Conversely, *PdeCRCK6* transcripts were induced under all treatments (PEG, mannitol, and ABA), showing consistent down-regulation after 24 h under PEG, an initial increase from 12 h to 24 h under mannitol, followed by a decline at 48 h and 72 h, and peaking at 12 h and 48 h under ABA. In all cases, expression at each time point was over 4-fold higher compared to 0 h (Figure 6a). All *PdeCRCKs* showed an induced expression following PEG treatment. The induction levels within 24 h for a short-term treatment under PEG was assessed, revealing distinct peak expressions of *PdeCRCKs*. Specifically, following PEG treatment, *PdeCRCK1* peaked at 1 h, *PdeCRCK3* at 12 h, and *PdeCRCK6* at 9 h and 12 h (Figure 6b). Additionally, three genes exhibited two peaks: *PdeCRCK2* peaked at 3 h to 6 h, *PdeCRCK4* at 1 h and 12 h, and *PdeCRCK5* at 1 h and 12 h (Figure 6b). These results suggest that the six *PdeCRCKs* may respond to osmotic stress in different ways.

### 2.7. Subcellular Localization and Potential Functions of PdeCRCK3/5/6

**Figure 7:**
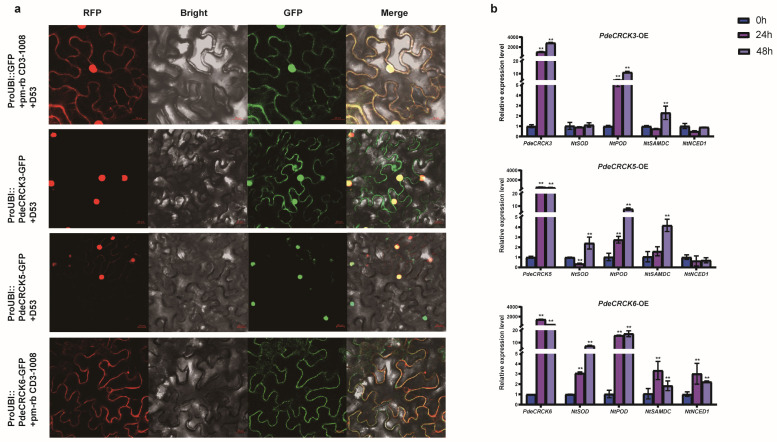
Subcellular localization and potential functions of PdeCRCK3, PdeCRCK5, and PdeCRCK6 in tobacco. (**a**) Subcellular localization of green fluorescent protein (GFP)-PdeCRCK3/5/6 fusion proteins in tobacco cells. Scale bar = 20 μm. D53 (DWARF 53) acts as a nuclear localization marker, and pm-rb CD3-1008 acts as a membrane-localization marker. (**b**) The relative expression levels of *PdeCRCK3/5/6* and some stress-responsive genes (*NtSOD*, *NtPOD*, *NtSAMDC*, and *NtNCED1*) when *PdeCRCK3/5/6* were overexpressed in tobacco at 0 h, 24 h and 48 h. Quantitative analyses of all measurements were conducted using GraphPad Prism 8. Data are presented as a mean ± standard deviation of three biological replicates. A 1.5-fold and 2-fold increase or decrease are denoted as * and **, respectively to indicate a statistically significant difference between the two conditions. The significance of differences was examined by Student’s *t*-test and Duncan’s multiple range test.

To investigate the gene function of PdeCRCKs, we determined the subcellular localization of three PdeCRCKs (PdeCRCK3, PdeCRCK5, and PdeCRCK6) in young tobacco leaf epidermal cells. The results show that the fluorescent signal of PdeCRCK3-GFP was mainly detected in the nucleus and cell membrane, PdeCRCK5-GFP is mainly localized in the nucleus, and PdeCRCK6-GFP is mainly localized in the cell membrane (Figure 7a). These results suggest that PdeCRCK3, PdeCRCK5, and PdeCRCK6 might have different functions.

To further investigate the relationship between *PdeCRCKs* and stress-responsive genes, the expression levels of several stress-responsive genes in tobacco were determined using qRT–PCR. These genes encode enzymes involved in reactive oxygen species (ROS) scavenging (*NtSOD* and *NtPOD*), ABA biosynthesis (*NtNCED1*), and polyamine synthesis (*NtSAMDC*). Compared to the empty EV6 in tobacco, a transient overexpression of *PdeCRCK3* resulted in a substantial increase, reaching 1054.18-fold at 24 h and further rising to 2844.78-fold at 48 h. Similarly, *PdeCRCK5*-OE led to an approximately 538.35-fold increase at 24 h and 452.89-fold increase at 48 h, and *PdeCRCK6*-OE expression increased approximately 1421.78-fold at 24 h and 394.50-fold at 48 h compared to the EV6 transient leaves. In leaves overexpressing *PdeCRCK3* (*PdeCRCK3*-OE), the expression levels of *NtPOD* and *NtSAMDC* were significantly elevated compared to those in leaves of the empty vector control (EV6), whereas *NtSOD* and *NtNCED1* showed no significant alterations. In *PdeCRCK5*-OE leaves, the expressions of *NtSOD*, *NtPOD*, and *NtSAMDC* were all significantly increased at 48 h, whereas *NtNCED1* showed no changes. In *PdeCRCK6*-OE, the expression of stress-related genes was significantly induced at 24 h and 48 h (Figure 7b). Therefore, it was inferred that an overexpression of *PdeCRCK3*/*5*/*6* activated genes involved in ROS scavenging and polyamine synthesis genes, particularly affecting the ABA signaling pathway in *PdeCRCK6*-mediated stress responses.

### 2.8. Proteins That Interact with PdeCRCK6

**Figure 8:**
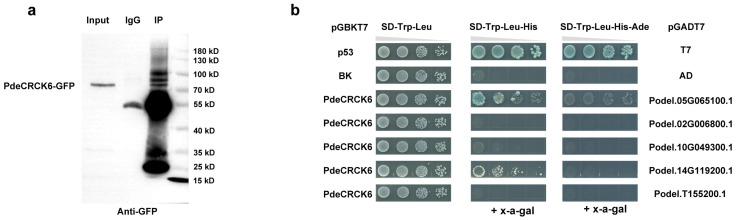
Proteins that interact with PdeCRCK6. (**a**) Co-immunoprecipitation of PdeCRCK6-GFP. The antibody is the ProteinFind Anti-GFP Mouse Monoclonal Antibody from TransGen Biotech. (**b**) PdeCRCK6 interacted with both AAA-type ATPase family proteins (Podel.05G065100.1) and ubiquitin 6 (Podel.14G119200.1), as determined by a yeast two-hybrid analysis.

Based on the above results, PdeCRCK6 may respond to osmotic stress by modulating the ABA signaling pathway, which is involved in ROS scavenging and polyamine synthesis. Therefore, *PdeCRCK6* was selected for further analysis. The full-length cDNA sequence of PdeCRCK6 was obtained, and a *ProActin*::PdeCRCK6-GFP vector was constructed and subsequently introduced into the leaves of ‘Danhong’ *Populus*. Immunoprecipitation followed by mass spectrometry (IP–MS) was employed to identify proteins interacting with PdeCRCK6 in vivo. An anti-GFP antibody was used to analyze input proteins from positive transgenic plants and IP extracts, suggesting that the IP product was suitable for an MS analysis. Following a peptide analysis of the MS data for PdeCRCK6, seven unique peptides were identified compared to a control IgG (Figure 8a). Among the proteins identified through IP, these peptides were found to encode glyceraldehyde-3-phosphate dehydrogenase B subunits, AAA-type ATPase family proteins, glyceraldehyde-3-phosphate dehydrogenase C subunit 1, glucose–methanol–choline (GMC) oxidoreductase family proteins, ubiquitin 6, and GDSL-like Lipase/Acylhydrolase superfamily proteins (Table 2).

To confirm the potential physical interactions between PdeCRCK6 and the identified interacting proteins, a Y2H assay was performed. While two of the genes were not successfully amplified, the cDNA from the other five genes was amplified and cloned into the pGADT7 vector, and PdeCRCK6 was cloned into the pGBKT7 vector. Then, five pairs of plasmids were co-transformed into yeast Y2H gold cells and grown on a synthetic dropout medium. The yeast colonies were subsequently tested on an SD/-Trp-Leu-His and SD/-Trp-Leu-Ade-His medium containing X-α-Gal. The results of the Y2H assay demonstrate that PdeCRCK6 interacted with both AAA-type ATPase family proteins (Podel.05G065100.1) and ubiquitin 6 (Podel.14G119200.1). The homologous gene of *Podel.05G065100.1* in *A. thaliana* was *AtFtsHi4* (Filamentation-Temperature-Sensitive Protein H) [37], a mutation of which leads to abnormalities in cotyledon growth, phenotype, and chloroplast ultrastructure. These results suggest that PdeCRCK6 could interact with Podel.05G065100.1, which is involved in chloroplast developments to mediate responses to abiotic stress (Figure 8b).

## 3. Discussion

The RLCK family, which lacks the extracellular domain, belongs to the RLK protein kinase superfamily that comprises all of the protein kinases identified in eukaryotes [38]. Calcium-binding receptor-like cytoplasmic kinases (CRCKs), also known as the receptor-like cytoplasmic kinases-IV (RLCK-IV) subfamily, function in post-translational protein modifications through their phosphorylation activity, a feature often reported in the analysis of RLK or RLCK family members. However, a comprehensive analysis of the RLCK-IV/CRCK subfamily members in plants remains insufficient. In this study, we identified six RLCK-IV subfamily genes in *P. deltoides*, which were distributed across five chromosomes. Most of the PdeCRCKs were located in the nucleus and cell membrane, as supported by PdeCRCK3 being expressed in the PM, cytoplasm, and nucleus and PdeCRCK6 being expressed in PM, which suggests that PdeCRCKs may function as an intermediate signal that travels from the cytoplasm to the nucleus. Conserved protein domains’ alignment of PdeCRCKs indicated that the kinase catalytic domain, encompassing the CaM-binding domain and the Ser/Thr phosphorylation site, were conserved, which plays an important role in modulating the interaction between the calcium/calmodulin sensor protein or the protein kinase activity. The exon–intron structures with conserved kinase catalytic domains were found in all PdeCRCKs, suggesting that PdeCRCKs have conserved functions within the RLCK-IV subfamily.

A phylogenetic analysis confirmed that most of the PdeCRCKs are more closely related to RLCK-IV genes of the dicot plant *A*. *thaliana*, suggesting similar functions during plant growth and development. As previously reported, most plant species have undergone large-scale whole-genome duplication events, including segmental duplication and tandem duplication, which have contributed to a larger genome size. In comparison with previous studies, there are three RLCK-IV subfamily genes in *A*. *thaliana* [10], four RLCK-IV subfamily genes in rice [23], twenty RLCK-IV subfamily genes in wheat [39], eight RLCK-IV subfamily genes in maize [40], and various genes identified in different plants, suggesting that whole-genome duplication events and tandem duplication are the main drivers behind the large differences in gene number in species. A collinear analysis of the *P. deltoides* genome, in comparison with rice and *A*. *thaliana*, revealed that the synteny relationship between *P. deltoides* and *A*. *thaliana* is stronger than that between *P. deltoides* and rice, suggesting a closer genetic relationship between dicots than between monocots and dicots.

RLCKs have been reported to modulate plant cell activities in response to abiotic stresses and endogenous extracellular signaling molecules. In *A*. *thaliana*, the RLCK-IV member AtARCK1 interacts with CRK36 (a cysteine-rich repeat RLK) to mediate ABA and osmotic-stress signaling pathways during post-germinative growth. In rice, a cytoplasmic receptor-like kinase, OsRLCK311, contributes to salinity tolerance by regulating ABA-dependent stomatal responses [41]. It was speculated that the members in poplar might possess similar functions in responding to osmotic stress via the ABA signaling pathway. In *P. deltoides*, six PdeCRCKs displayed diverse numbers of *cis*-elements and distinctive expression patterns under ABA, mannitol, and PEG stresses. The expression pattern of *PdeCRCK3* suggests that with a continuous exposure to PEG and an escalating ABA level in the plant, subsequently, the expression of *PdeCRCK3* was suppressed, thereby signifying its involvement in response to PEG via the ABA signaling pathway. In contrast, *PdeCRCK6* was significantly induced under all three stresses, indicating that it may respond to stress through ABA-dependent pathways. These results imply that *PdeCRCK3* and *PdeCRCK6* might function in different signaling pathways involved in osmotic stress.

Interaction proteins can provide powerful regarding protein functions and aid in elucidating potential molecular mechanisms. Numerous studies have demonstrated that chloroplast homeostasis assumes a crucial role in balancing plant growth and responses to environmental stress, with chloroplast proteins implicated in the repair of the photosystem and the degradation of damaged proteins via chloroplast protease systems, including Clp, FtsH, and Deg [42,43,44,45]. Nevertheless, the mechanism of its involvement with RLCK in regulating plant stress resistance has rarely been reported. In *P. deltoides*, we found that PdeCRCK6 interacted with Podel.05G065100.1, which is homologous to FtsHi4 in *A*. *thaliana*, potentially shedding light on PdeCRCK6’s role in regulating chloroplast homeostasis to adapt to abiotic stress. In *A*. *thaliana*, the T-DNA insertion mutant of *FtsHi4* led to an embryonic arrest at the globular-to-heart-shaped transition stage, accompanied by abnormal plastid differentiation and a severe defect in thylakoid formation within the mutant embryos [37]. The interaction results imply that PdeCRCK6 might regulate plant stress resistance by influencing the formation of chloroplast thylakoids and maintaining chloroplast homeostasis through the chloroplast degradation pathway. However, the functional mechanisms underlying the roles of PdeCRCKs in abiotic stress responses demand further investigation. Such studies could enhance our understanding of how plants grow in response to environmental stress, providing a foundation for molecular breeding strategies aimed at enhancing desirable traits in *P. deltoides*.

## 4. Materials and Methods

### 4.1. Identification of RLCK-IV Genes in P. deltoides

The genome-wide data for plant species including *P. deltoides Marsh*. (PdeltoidesWV94_445_v2.0)*, A*. *thaliana*, wheat, maize, and rice were obtained from Phytozome V13 (Phytozome (doe.gov), accessed on 11 January 2024), TAIR (TAIR-Home (arabidopsis.org), accessed on 11 January 2024), and Ensembl Plants (Ensembl Plants, accessed on 11 January 2024). The protein sequences of 3 AtRLCK-IVs [10] and 4 OsRLCK-IVs [23] were used as query templates to search for the homologous genes in *P. deltoides* by BLASTp. Proteins with identities of >50% and an E value of >1 × 10^−5^ were selected from the retrieved list for each query, in which proteins were arranged in descending order of their bit scores.

The resulting genes from all the queries were consolidated, and the protein sequences of putative RLCK-IV/CRCK subfamily members in *P. deltoides* were submitted to the NCBI Conserved Domain Database (NCBI-CDD) for conserved domain analyses (E value = 1 × 10^−2^). The transmembrane domain was predicted using TMHMM (https://services.healthtech.dtu.dk/services/TMHMM-2.0/, accessed on 27 February 2024), and SMART was employed to further confirm the motifs and exclude proteins that were not RLCK-IVs. The conserved domains were analyzed and visualized using Jalview 2.11.4.0.

### 4.2. Physicochemical Property Characterization of PdeCRCKs

The physicochemical characteristics of RLCK-IVs/CRCKs in *P. deltoides* were characterized using the “Protein Parameter Calc program” in TBtools 2.0 and ExPASy (https://web.expasy.org/protparam/, accessed on 5 March 2024). Subcellular localizations were predicted using Plant-mPLoc (http://www.csbio.sjtu.edu.cn/bioinf/plant-multi/, accessed on 10 March 2024). Multiple sequence alignment was performed using MEGA 11.0.13. The conserved domains were analyzed with the Conserved Domain Database (CDD) in NCBI and TBtools 2.0 [46].

### 4.3. Sequence Alignment and Phylogenetic Tree Construction

The RLCK-IV subfamily protein sequences of 4 OsRLCK-IVs [23], 8 ZmaRLCK-IVs [40], 3 AtRLCK-IVs [10], and 20 TaRLCK-IVs [39] and 3 PdeCRCKs were utilized to construct a phylogenetic tree using ClustalW. The best protein models were analyzed using MEGA 11.0.13. The optimal model for maximum likelihood (ML) trees was selected by MEGA 11.0.13. The phylogenetic relationship among the five plant species was analyzed using the maximum likelihood method (ML) and a bootstrap analysis with 1000 replicates in MEGA 11.0.13. The resulting phylogenetic tree was visually enhanced using Evolview (https://www.evolgenius.info/evolview/, accessed on 25 March 2024).

### 4.4. Gene Structure, Motif Identification, and Chromosomal Location

To investigate the structure of *PdeCRCKs*, TBtools 2.0 was used to visualize the exon–intron map based on the genome and GFF files. The ten conserved motifs were identified using MEME (https://meme-suite.org/, accessed on 10 April 2024) by a classic model and were depicted using TBtools 2.0. The chromosomal positions of *PdeCRCKs* were extracted from the genome and GFF files of *P. deltoides* and were visualized using the Gene Location Visualization module of TBtools 2.0. Additionally, the conserved motifs were visualized using WebLogo (WebLogo—Create Sequence Logos (berkeley.edu), accessed on 25 May 2024).

### 4.5. Cis-Acting Regulatory Elements’ Identification in Putative Promoter Sequences

The promoters (2000 bp upstream of transcriptional start site) of *PdeCRCKs* were extracted using TBtools 2.0 from the genome and GFF files. The *cis*-elements, including those responsive to stresses, hormones, and transcription factor binding sites in the promoters, were predicted using PlantCARE (a database of plant promoters and their cis-acting regulatory elements (ugent.be), accessed on 6 May 2024).

### 4.6. Synteny Relationships of RLCK-IV/CRCK Subfamily Members

To investigate the collinearity relationships among *P. deltoides*, *O. sativa*, and *A. thaliana*, the One Step MCScanX-Super Fast program in TBtools 2.0 was utilized for a preliminary analysis of intra-species and inter-species collinearity in *P. deltoides*. The Advanced Circos program in TBtools 2.0 was used to visualize the collinearity among RLCK-IVs/CRCKs [46].

### 4.7. Plant Materials, Drought Stress, RNA Extraction, and qRT–PCR Analysis

The tissue-cultured seedlings of ‘Danhong’ *populus* were grown under constant conditions of 25 °C with 16 h of light and 8 h of darkness. Tissue-cultured seedlings that grew consistently were selected for pre-culturing in a growth medium for 3 days. Then, these plant materials were treated with 600 mM of mannitol, 400 μM of ABA, and 10% PEG-6000. Leaves of ‘Danhong’ *populus* were collected at 0 h, 12 h, 24 h, 48 h, and 72 h after treatment initiation. To assess the response of the *PdeCRCKs* to a simulated drought stress over a short period, leaves were collected when tissue-cultured seedlings were treated with 10% PEG-6000 at 1 h, 3 h, 6 h, 9 h, 12 h, and 24 h.

### 4.8. Subcellular Localization and Transient Expression Analysis of PdeCRCKs in Tobacco

The CDS of *PdeCRCK3*, *PdeCRCK5*, and *PdeCRCK6* were cloned using the cDNA of ‘Danhong’ *populus*, and the CDS of these *PdeCRCKs*, without termination codons, were cloned into the *ProUbi::GFP* vector that is controlled by a UBI promoter to obtain the *ProUbi::PdeCRCK3/4/5/6-GFP* fusion vector with gene-specific primers (Table 3). The recombinant plasmid was then transformed into *Agrobacterium* GV3101, followed by a transient transformation of tobacco leaves. Gene localization in cells was observed, and leaf samples were collected at 0 h, 24 h, and 48 h after transformation. D53 (DWARF 53) [47] was used as a nuclear localization marker, and pm-rb CD3-1008 was used as a membrane-localization marker [48].

### 4.9. Quantitative qRT–PCR Analysis

The total RNA was extracted using the Tiangen RNAprep plant kit (Tiangen, Beijing, China), and the first-strand cDNA was synthesized using the ReverTra Ace qPCR RT Kit (Toyobo, Fukui, Japan). Quantitative real-time PCR was performed using TB Green Premix Ex Taq (TAKARA, Beijing, China) with gene-specific primers on a QuantStudio^TM^7 Flex Real-Time PCR instrument (Applied Biosystems, Foster City, CA, USA). RG5 [49] was used as an internal control in ‘Danhong’ *populus*, and ubiquitin [50] was used as an internal control in tobacco to normalize gene expressions. The primer sequences used in the qRT–PCR analysis are detailed in Table 3 [50].

### 4.10. Immunoprecipitation–Mass Spectrometry (IP–MS)

A full-length coding sequence of PdeCRCK6 was cloned into the *pCAMBIA2300-Actin*::*GFP* vector and subsequently transformed into the leaves of ‘Danhong’ *Populus*. An anti-GFP antibody was used to identify positive transgenic plants. Forty-day-old transgenic seedlings of *P. deltoides* expressing *ProActin::PdeCRCK6-GFP*, cultivated under 16 h of light and 8 h of darkness at 25 °C, were harvested. To facilitate the formation of immune complexes, we co-incubated the ProActin::PdeCRCK6-GFP total protein with the PdeCRCK6-GFP protein as a bait. Protein A/G, which binds to the Fc region of the anti-GFP mouse monoclonal antibody (TransGen Biotech, Beijing, China), was introduced to form ‘bait protein-target protein-target protein antibody-protein A/G beads’ complexes, which allowed the proteins interacting with PdeCRCK6-GFP to be co-precipitated. Finally, the purified complexes were separated by SDS-PAGE. The target protein strips were cut off and rinsed twice with ultrapure water, followed by decolorization and dehydration. After the removal of water, the dehydrated gels were freeze-dried under a vacuum. The freeze-dried gel pieces were blocked with a reducing solution, and the enzymolysis supernatant was obtained [51].

The nano-HPLC liquid chromatography UltiMate 3000 RSLCnano (ThermoFisher Scientific, Waltham, MA, USA) was used for sample separation. Dried polypeptide samples were re-dissolved and subsequently loaded by an automatic sampler onto a trap column, where they were separated. The samples were cleaned by a mobile phase gradient with a blank solvent and were separated by capillary high-performance liquid chromatography and were analyzed by Q-Exactive Plus mass spectrometry (ThermoFisher Scientific).

MS/MS spectra were searched using Proteome Discover 2.5 against the PdeltoidesWV94_445_v2.1 protein FASTA database. The search parameters were set as follows: static modifications: Carbamidomethyl (C); dynamic modifications: Acetyl (Protein N-term), Deamidated (NQ), and Oxidation (M); first-search peptide tolerance: 10 ppm; main-search peptide tolerance: 0.05 Da; max missed cleavages: 2.

### 4.11. Yeast Two-Hybird Assays

The full-length coding sequences of *PdeCRCK6* and *Podel.02G006800.1*, *Podel.04G229700.1*, *Podel.05G065100.1*, *Podel.10G049300.1*, and *Podel.14G119200.1* were amplified using the cDNA of *P. deltoides* and were fused to the GAL4 DNA binding domain of pGBKT7 and the GAL4 activation domain of pGADT7 to generate a prey vector and a bait vector. The combinations of bait and prey constructs were co-transformed into the yeast strain Y2H and were selected on an SD medium lacking leucine (Leu) and tryptophan (Trp) for 4 days at 30 °C. Yeast cells were plated on selection plates containing an SD medium lacking Trp, Leu, Ade (adenine), and His (histidine) for an interaction test. The primers are listed in Table 3.

### 4.12. Quantification and Statistical Analysis

Quantitative analyses for all measurements were performed using GraphPad Prism 8. Data are presented as a mean ± standard deviation (SD). Statistical significance was assessed using Students’ *t*-test and Duncan’s multiple range test: ^ns^ *p* > 0.05, * *p* < 0.05, ** *p* < 0.01.

## 5. Conclusions

In this study, we identified six *PdeCRCK* genes belonging to the RLCK-IV subfamily in *P. deltoides*, which are located on 5 chromosomes. A comprehensive analysis encompassing evolution, conserved domains and motifs, gene structure, chromosome distribution, synthesis, *cis*-acting elements, and responses to abiotic stresses and hormones was constructed. Notably, PdeCRCKs had conserved kinase catalytic domains and functions within RLCK-IV subfamily proteins. Segmental duplication appears to be the primary force driving the expansion of the PdeCRCK gene family. The expression of *PdeCRCK6* was found to respond to the osmotic stresses in an ABA-dependent pathway. PdeCRCK6 interacted with AAA-type ATPase proteins, suggesting that PdeCRCK6 may be involved in regulating chloroplast homeostasis to adapt to osmotic stress. Overall, these findings enhance our understanding of the RLCK-IV subfamily in *P. deltoides*, and the potential function of PdeCRCK6 in osmotic stress may provide a key target and an important theoretical basis for accelerating the molecular breeding of drought tolerance in poplar.

## Figures and Tables

**Table 1:** Basic information about RLCK-IV/CRCKs subfamily members in *Populus deltoides*.

Gene Name	Gene Locations	CDS (bp)	Protein Length (aa)	MW (kDa)	PI	Instability Index	Aliphatic Index	Sub Location
*PdeCRCK1*	Podel.*02G178500.1.v2.1*	1171	389	43,623	8.78	45.49	83.5	Nucleus
*PdeCRCK2*	Podel.*02G180200.1.v2.1*	1324	440	49,046	8.59	50.25	76.7	Nucleus
*PdeCRCK3*	Podel.*06G069100.1.v2.1*	1527	508	56,946	7.94	39.78	90.93	Cell membrane and nucleus
*PdeCRCK4*	*Podel*.*09G041200.1.v2.1*	1356	451	49,881	9.49	41.82	78.69	Nucleus
*PdeCRCK5*	*Podel*.*14G089100*.*1.v2.1*	1320	439	48,644	9.24	49.59	81.12	Nucleus
*PdeCRCK6*	*Podel.18G126600.1.v2.1*	1542	513	57,581	7.99	38.31	93.1	Cell membrane

**Table 2:** Annotation of 7 interaction proteins with PdeCRCK6 by IP–MS.

ID	Gene Id	Pfam	GO	Best-Hit-Arabi	Arabi-Defline
1	*Podel.02G006800.1*	PF02672, PF00044, and PF02800	GO:0055114 and GO:0016620	*AT1G42970.1*	Glyceraldehyde-3-phosphate dehydrogenase B subunit
2	*Podel.04G229700.1*				
3	*Podel.05G065100.1*	PF05496	GO:0009378, GO:0006310, and GO:0006281	*AT5G64580.1*	AAA-type ATPase family proteins
4	*Podel.10G049300.1*	PF00044 and PF02800	GO:0055114 and GO:0016620	*AT3G04120.1*	Glyceraldehyde-3-phosphate dehydrogenase C subunit 1
5	*Podel.12G142900.1*	PF05199 and PF00732	GO:0055114, GO:0016614, and GO:0050660	*AT5G51950.1*	Glucose–methanol–choline (GMC) oxidoreductase family proteins
6	*Podel.14G119200.1*	PF00240 and PF01599	GO:0005515 and GO:0006412, GO:0005840, and GO:0003735	*AT2G47110.2*	Ubiquitin 6
7	*Podel.T155200.1*	PF00657	GO:0016788	*AT1G29670.1*	GDSL-like Lipase/Acylhydrolase superfamily proteins

**Table 3:** The primer sequences used in this study.

Gene ID	Primer Sequence F (5′-3′)	Primer Sequence R (5′-3′)
*PdeRG5*-RT	CCCAGAGCCGCACCAACT	TGGGTTTCTTGATGCCATTTTG
*Podel.06G209700.1*-RT	AAGCCTCCGGAGCAAATGAA	CCTGCACTTGGTGTCCTCTT
*Podel.02G016400.1*-RT	ATGCATCGGCACAGACTTGA	TCATGCTCGCAAACTCCTCA
*Podel.16G071000.1*-RT	TGGGTCCTTGACTAAAGTGC	GAGGCTTGTTTGGTCTTGCG
*Podel.01G118600.1*-RT	GGGCATGATGGGAAGTGGAA	GGTCTTGCCTTTCATGGGGA
*Podel.05G261000.1*-RT	ACCAACCAACACCACCTATACC	TTCCCGACGCCTTCTCTGTA
*PdeCRCK1*-RT	TTTTGTCTTTTGCCCACGCT	GCCTCGATTTGAACCTTGCC
*PdeCRCK2*-RT	TTCTGGGAGCAGTGAAAGAAGG	CACCTTGTCCAATCTTGAATGTG
*PdeCRCK3*-RT	ACCCCCATGAGTGACGTGTA	ACCTCCGTGTCCACCTTTTC
*PdeCRCK4*-RT	TGCGAGATTGACCACCGAAT	TCAATCGGGTGTCTTCCTGT
*PdeCRCK5*-RT	GTAGGCGCCCTATTGAAGCA	GCCAAGTTATTTGCTGCGGT
*PdeCRCK6*-RT	GCGCCCTGTGGAGATGAAG	TACACCTTTCAGGTAGTCTGCT
*NtUBQ*-RT	TCCAGGACAAGGAGGGTAT	CATCAACAACAGGCAACCTAG
*NtSOD*-RT	AGCTACATGACGCCATTTCC	CCCTGTAAAGCAGCACCTTC
*NtPOD*-RT	AAATGGTGGCGCTAGCCGGTG	GCATTGAAGACGTGCCGCTGG
*NtSAMDC*-RT	CATTCACATTACCCCGGAAG	AGCAACATCAGCATGCAAAG
*NtNCED1*-RT	AAGAATGGCTCCGCAAGTTA	GCCTAGCAATTCCAGAGTGG
PdeCRCK3-GFP	gtgttacttctgcaggagctcATGGCCACGGCTGCATT	catggatccggtaccgagctcCCCTTTCTTTACACCTTTCAG
PdeCRCK5-GFP	gtgttacttctgcaggagctcATGAAGAGCCCATATTC	catggatccggtaccgagctcTTCTTCTGTTATTACTGAAAT
PdeCRCK6-GFP	gtgttacttctgcaggagctcATGGCCACGGCTGCATT	catggatccggtaccgagctcCCCTTTCTTTACACCTTTCAG
PdeCRCK6-2300	cggggatcctctagagtcgacATGGCTATGAATGCATT	tccggtaccgagctcgtcgacCCCTTTCTTTACACC
PdeCRCK6-BD	tggccatggaggccgaattcATGGCTATGAATGCATT	cgctgcaggtcgacggatccTTACCCTTTCTTTACACC
Podel.02G006800.1-AD	gccatggaggccagtgaattcATGGCCACCCACGCAG	atgcccacccgggtggaattcCTAAGCTTCATAGACTTTGC
Podel.04G229700.1-AD	gccatggaggccagtgaattcATGAACATTGATAGAC	atgcccacccgggtggaattcTCAAGGACGTACGATAGCA
Podel.05G065100.1-AD	gccatggaggccagtgaattcATGAAATCCCTCGTTTC	atgcccacccgggtggaattcTCAAAGGAAATGGCTGGCC
Podel.10G049300.1-AD	gccatggaggccagtgaattcATGGCATGTGATAAGA	atgcccacccgggtggaattcTCAAGCTTGAGTCTTGGCC
Podel.14G119200.1-AD	gccatggaggccagtgaattcATGGCTAAATCCGTACT	atgcccacccgggtggaattcTTAATCACCACCAGCCGTC

## Data Availability

Data are contained within the article.
